# Transcription factors in fibroblast plasticity and CAF heterogeneity

**DOI:** 10.1186/s13046-023-02934-4

**Published:** 2023-12-20

**Authors:** Roberta Melchionna, Paola Trono, Anna Di Carlo, Francesca Di Modugno, Paola Nisticò

**Affiliations:** 1 Tumor Immunology and Immunotherapy Unit, IRCCS-Regina Elena National Cancer Institute, Rome, Italy; 2 Institute of Biochemistry and Cell Biology (IBBC), National Research Council (CNR), Rome, Italy

**Keywords:** Cancer Associated fibroblasts (CAFs), Transcription factors (TFs), CAF activation, CAF subtypes, Fibrosis

## Abstract

In recent years, research focused on the multifaceted landscape and functions of cancer-associated fibroblasts (CAFs) aimed to reveal their heterogeneity and identify commonalities across diverse tumors for more effective therapeutic targeting of pro-tumoral stromal microenvironment. However, a unified functional categorization of CAF subsets remains elusive, posing challenges for the development of targeted CAF therapies in clinical settings.

The CAF phenotype arises from a complex interplay of signals within the tumor microenvironment, where transcription factors serve as central mediators of various cellular pathways. Recent advances in single-cell RNA sequencing technology have emphasized the role of transcription factors in the conversion of normal fibroblasts to distinct CAF subtypes across various cancer types.

This review provides a comprehensive overview of the specific roles of transcription factor networks in shaping CAF heterogeneity, plasticity, and functionality. Beginning with their influence on fibroblast homeostasis and reprogramming during wound healing and fibrosis, it delves into the emerging insights into transcription factor regulatory networks. Understanding these mechanisms not only enables a more precise characterization of CAF subsets but also sheds light on the early regulatory processes governing CAF heterogeneity and functionality. Ultimately, this knowledge may unveil novel therapeutic targets for cancer treatment, addressing the existing challenges of stromal-targeted therapies.

## Background

Tissue-resident fibroblasts are mesenchymal cells that possess an impressive plasticity in their ability to functionally adjust their properties according to the requirements of the microenvironment. There are diverse subgroups of fibroblast phenotypes associated with different tissue pathological conditions, e.g., wound healing, many fibrotic and inflammatory conditions, and cancer [1].

In the tumor microenvironment (TME) CAFs are the most abundant stromal cells and were recently positioned at the top of a hierarchical network of cell interactions [2]. Crucially involved in different hallmarks of cancer tumorigenesis, cancer progression, metabolism and immune response, CAFs promote immune evasion, tumor metastasis, and therapy resistance by remodeling the extracellular matrix (ECM), secreting growth factor and cytokines [3–6]. However, it is largely accepted that different subpopulations of CAFs, with context-dependent pro-or antitumor activities, can coexist in the TME of different solid tumors [7].

Transcription factors (TFs) act as a key hub for fibroblast homeostasis and may exert a crucial regulatory role in conversion from normal fibroblasts (NFs) into CAFs activation and commitment towards different subsets by converging both CAF-intrinsic and extrinsic signaling pathways. Recent single-cell RNA sequencing (scRNA-seq) studies in several tumors have revealed an enrichment of gene regulatory networks of TFs in the CAF subtypes [8, 9], supporting the role of TFs in regulating the heterogeneous CAF functionality.

Overall, the identification of specific transcriptional networks governing the transition of NFs into CAF and defining CAF subset specification would represent an appropriate approach towards a unified functional definition of CAF subtypes. Additionally, novel therapeutic approaches targeting CAF subset specific TFs would enable the development of specific stromal-directed therapies for cancer.

This review aims to discuss how the identification of master TFs may provide an opportunity for the definition of CAF subsets, their functional role in cancer progression and likely cancer treatment. Starting with the role of TFs in regulating NF homeostasis, fibroblast reprogramming in wound healing and fibrotic fibroblasts, we describe the contribution of specific TFs to NF conversion into CAFs, and their role in the regulation of phenotypic and functional heterogeneity of CAFs. Finally, we discuss the potential of TF-targeted approaches as an anticancer therapeutic strategy.

## Transcription factors in fibroblast homeostasis, wound healing and fibrosis

**Fig. 1:**
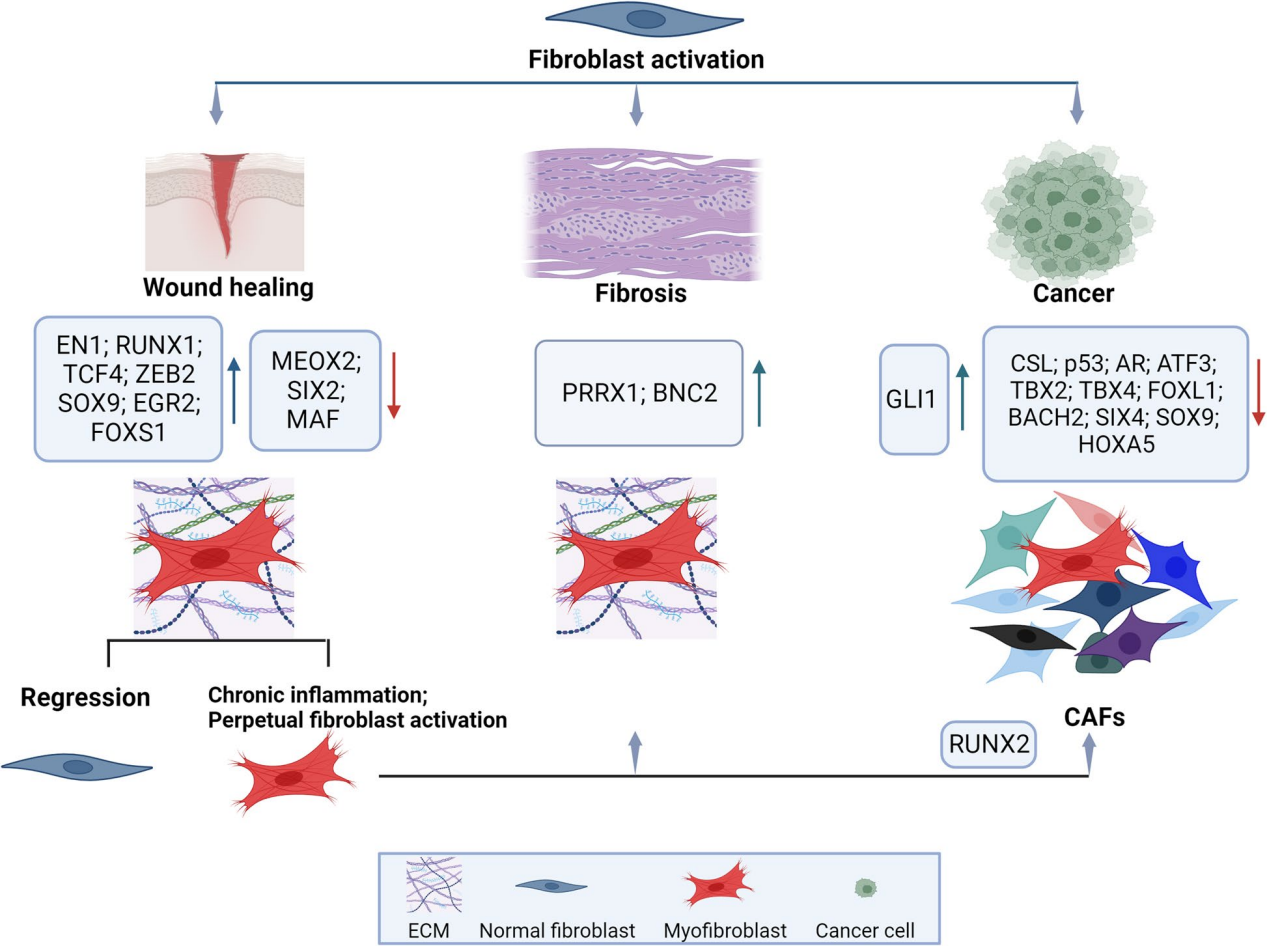
Schematic representation of the main transcription factors involved in normal fibroblasts conversion into myofibroblasts during wound healing, fibrosis and cancer. EN1-positive dermal fibroblasts are major contributors toward wound repair. The major TFs upregulated or downregulated during fibroblast activation due to wound healing, fibrosis or cancer are reported. RUNX2 identifies both wound fibroblasts and the “early wound CAF” subtype. CSL complex with p53, ATF3 or AR to act as transcriptional repressor control of early CAF activation. Created with BioRender.com

Fibroblasts are the most abundant cell types in connective tissues, responsible for tissue homeostasis under normal physiological conditions and their activation is a critical event in wound healing, chronic fibrotic diseases and cancer (Fig. 1). When tissues are injured, in the early proliferative phase of wound healing, fibroblasts become activated and differentiate into myofibroblasts, which actively produce ECM proteins and play roles in inflammation and immune cell recruitment to sites of tissue injury to facilitate wound closure [10]. Once the wound is repaired, the number of fibroblasts markedly decreases and results in the restoration of fibroblast tissue homeostasis. This regulation is disturbed during chronic inflammation that leads to a non-healing pattern and tissue damage rather than repair due to a perpetual fibroblast activation, which induces fibrosis. A similar landscape occurs in the TME where chronic CAF activation promotes cancer progression [11]. Along these lines, more than three decades ago, tumors have been described as a wound that does not heal [12] indicating that in response to pathological cues, physiological processes such as wound healing can become detrimental [13].

TFs play a key role in controlling the gene regulatory networks, by recognizing and directly binding to specific DNA promoter regions. Their activity also results in the recruitment of co-factors, enabling RNA polymerase II complex to perform mRNA transcription at gene enhancers and promoters. The ability of TFs to redistribute from the cytoplasm to the nucleus is also critical for their proper function as signaling molecules.

Normal fibroblasts activation into myofibroblasts during wound healing and their conversion into normal fibroblasts once the wound is healed have strongly supported the concept of fibroblast plasticity. Different TFs have been involved in the fibroblasts switching to myofibroblasts. Among these, c-JUN and c-FOS, for activator protein-1 (AP-1), SMAD2/3 for TGF-β signaling, β-catenin for wingless type (Wnt) signaling and the MRTF-SRF signaling axis [10, 14].

The study of the dynamic diversity of wound fibroblasts during 12-days post wound healing, by single-cell RNA-sequencing analysis of murine skin, recently revealed that wounding induces a high degree of heterogeneity among fibroblasts. Wound fibroblasts were grouped into twelve subclusters [15] with a shared expression of twenty TFs, defined as wound fibroblast TF signature, which included Runt-related transcription factors 1 (RUNX1), Transcription Factor 4 (TCF4), and Zinc-finger E-box-binding homeobox 2 (ZEB2), also implicated in myofibroblast differentiation. The Authors were able to broadly classify the wound fibroblasts into two major populations based on their transcription factor signatures and PDGF receptor expression pattern (Fig. 1 and Table 1).

**Table 1:** Transcription factors grouped by the biological process of action

Transcription factors	Key observations	Reference
**Wound healing and fibrosis**		
Engrailed-1 (EN1)	A major player in wound repair, contributes to the scarring process	[16, 17]
c-Jun/c-fos	Drive AP-1 activation during wound healing in neonatal and adult skin	[10, 18]
SMAD2/3	Downstream to TGF-β signaling activation in fetal and adult wound healing process	[10, 19]
β-catenin	Downstream to Wingless type (Wnt) signaling during wound tissue remodeling	[10, 20]
RUNX1, TCF4, ZEB2	“Wound fibroblast TF signature” contributing to the wound healing process	[15]
TCF4, SOX9, EGR2, FOXS1	Drive myofibroblast differentiation in chronic wounds	[21]
LEF1	Promotes healthy skin regeneration in young skin	[22]
ZFP423	Drives regeneration of fat cells from myofibroblasts during wound healing	[23]
PRRX	Drives a pro-fibrotic response in idiopathic pulmonary fibrosis	[24]
BNC2	Sustains the myofibroblastic activation in liver fibrosis	[25]
**Normal fibroblast conversion into CAFs**		
GLI1	Specific Gli1 + fibroblasts expansion in tumor stroma during carcinogenesis	[26]
TBX4	Lost during lung CAF activation Promotes fibroblast proliferation and collagen gel contraction capacity	[27]
CSL/p53 complex	Lost during early CAF activation Direct repressor of CAF-effector genes. Repressor of p53	[28]
ATF3	Lost during early CAF activation Converges with CSL complex to inhibit CAF-determining genes	[29]
Androgen receptor	- Lost during early CAF activation. Converges with CSL complex to repress key CAF effector genes	[30]
	- AR loss promotes the tumor-promoting abilities of CAFs	[31]
	- AR loss induces deformation of nuclear shape, and nuclear abnormalities and inhibits CAF features	[32]
SMAD2/3	Sustains TGF-β and SDF-1 autocrine signaling required for NF conversion into CAFs	[33]
HSF1	Sustains TGF-β and SDF-1 autocrine signaling	[34]
RUNX3/MYC	Sustains TGF-β autocrine signaling	[35]
YAP-TEAD	Downstream to mechanotransduction and matrix remodeling sustain CAF generation and maintenance	[36]
HSF1/ Dickkopf-3	Positive regulators of YAP nuclear translocation and activation of target gene	[37]
MRTF-SRF	Crosstalk with YAP-TEAD signaling. Downstream to mechano-transduction, induce CAF contractile and pro-invasive properties	[38]
SNAIL1	Downstream to YAP-mediated mechano-transduction Induces fibronectin and collagen expression and promotes matrix rigidity	[39]
ZNF416	Downstream to mechano-transduction, supports fibroblast contractile activation, proliferation, and ECM synthesis	[40]
HIF-1α	Drives metabolic reprogramming in breast cancer cells leading to CAF activation	[41]
POU1F1	Drives metabolic reprogramming of both CAFs and cancer cells	[42]
c-FOS and c-JUN	Modulate the expression of glycolytic enzymes required for CAF activation	[43]
TFAM	Its downregulation in CAFs induces mitochondrial dysfunction and metabolic reprogramming towards aerobic glycolysis promoting tumor cell growth	[44]
RUNX1	Sustains mesenchymal stem cell differentiation into myofibroblasts in prostate cancer stroma	[45]
ZNF32	When expressed in breast tumor cells, leads to CAF transformation from normal fibroblasts	[46]
**CAF activation and pro-tumoral functions**		
SNAIL1	- Sustains CAF activation and pro-tumoral functions across various cancers	[47]
	- Regulates fibroblast activation protein alpha (Fap) expression and promotes immune suppression in melanoma	[48]
TWIST	Sustains Twist1-Prrx1-TNC positive feedback loop	[49]
PRRX1	When depleted, forces CAFs into a highly activated state with increased ECM deposition	[50]
ZEB1	Sustains pro-tumoral CAF features	[51]
RUNX2	Sustains pro-tumoral CAF functions in bladder cancer	[52]
RUNX1	Sustains early activation of CAF-tumor cell crosstalk	[53]
p53	Activates late stage of CAF-specific genes	[54]
ATF3	Activates late stage of CAF-specific genes	[55]
STAT-3	Paracrine pro-tumorigenic CAF functions in breast cancer	[56]
**CAF plasticity and heterogeneity**		
RUNX2	Regulates “early wound CAF” subtype signature	[57]
FOX TFs	Increased activity in precancerous adenomas “intermediate state” during transformation from healthy to colorectal cancer	[58]
RUNX1	Increased activity in cancerous state of colorectal cancer	[58]
MYC	Sustains metastasis-associated fibroblast rewiring in lung cancer	[59]
ZEB1	- Promotes myofibroblastic features of colorectal cancer-derived CAFs	[51]
	- Sustains CAF reprogramming via a secretory program	[60]
PRRX	- Acts as master TFs of stromal fibroblasts for myofibroblastic lineage progression in multiple cancer types	[61]
	- Induces CAF activation in PDAC, allowing a dynamic switch between a dormant and an activated state	[50]
SALL4	Sustains TGF-β-activated CAF subsets in PDAC	[62]
SMAD2	Defines TGF-β-activated myofibroblasts	[33]
SOX2	Drives colonic fibroblasts reprogramming and promotes pro-tumoral myofibroblast functions and immunosuppressive tumor microenvironment	[63]
**CAF plasticity and heterogeneity mediated by cancer cell contextual cues**		
ETV1	- Sustains inflammatory iCAF features. Controls the duality of FGF/TGF-β signaling in skin squamous cell carcinomas	[64]
	- Controls TGF-β /HGF and FGF7 signalling in non-small cell lung cancer	[65]
STAT3	Sustains inflammatory iCAF features induced by tumor-derived IL-1 in naïve pancreatic stellate PDAC cells	[66, 67]
SMAD2	Sustains myCAF features induced by tumor-derived TGF-β in naïve pancreatic stellate PDAC cells	[66, 67]
MZF1	Sustains the mesenchymal stem cells to-myCAF conversion in breast cancer	[68]
RUNX1	Associated with specific TFs network involved in pro-tumoral cancer cell/CAF crosstalk in prostate cancer	[53]
ZEB1	Its expression in tumor cells reprograms CAFs to promote metastasis in lung adenocarcinoma	[46, 60]
ZNF32	Its expression in tumor cells prevents fibroblast activation in breast cancer cells	[46]
P53	Its mutational status in pancreatic cancer cells drives CAF hierarchy to establish a pro-metastatic and chemoresistant TME	[69]

By establishing a human ex vivo model of chronic wounds, a TF network which includes TCF4, SRY-Box Transcription Factor 9 (SOX9), Early growth response 2 (EGR2), and Forkhead Box S1 (FOXS1) has been defined as major regulator of fibroblast to myofibroblast differentiation. This study also identified a TF network essential for cellular reprogramming which includes Mesenchyme Homeobox 2 (MEOX2), SIX Homeobox 2 (SIX2), and MAF bZIP transcription factor (MAF), whose downregulation leads to a TGF-β-independent reprogramming of fibroblasts to myofibroblasts [21] (Fig. 1 and Table 1).

The involvement of TF in programming fibroblasts to participate in tissue repair emerges also by recent data indicating that Lymphoid enhancer-binding factor 1 (LEF1)-positive fibroblasts primed the skin microenvironment to enhance skin repair [22] and Zinc Finger Protein 423 (ZFP423) activation during wound healing drives myofibroblasts to adipogenic lineage commitment [23].

Lineage tracing study identified Engrailed-1 (EN1)-positive dermal fibroblasts as major contributors toward wound repair by scarring [16]. The postnatal inhibition of EN1 activation, either directly (by ablating EN1-activating cells) or indirectly (by blocking mechanical signaling), has been shown to promote skin regeneration without scarring by EN1 lineage-negative fibroblasts [17] (Fig. 1 and Table 1).

Interestingly, that wound fibroblasts and CAFs share signatures has been recently demonstrated in an elegant paper where RUNX2 was identified as crucial to define early wound CAF subtype signature, as discussed later [57] (Fig. 1 and Table 1).

Noteworthy dysregulation of transcriptional networks that govern fibroblast homeostasis and functions is also required for the aberrant phenotypic changes of fibroblasts during tissue fibrosis.

In idiopathic pulmonary fibrosis, the Paired-related Homeobox Protein (PRRX1) TF has been identified as crucially involved in eliciting a pro-fibrotic response [24]. PRRX1 contributes not only to the maintenance of lung mesenchymal cells in an undifferentiated and proliferative state, but also promotes TGFβ-mediated myofibroblastic differentiation. A multi-omics analysis has evidenced the crucial role of the transcription factor Basonuclin 2 (BNC2) in the activation of canonical pathways driving myofibroblastic activation in the context of liver fibrosis. The authors found that BNC2 transcriptional induction is a specific feature of in vivo myofibroblastic activation in liver fibrosis, extended to lung or heart injury, indicating BNC2 as a marker of myofibroflasts across different organs. Through both its expression and its transcriptional regulatory activities, BNC2 controls the expression of matrisome genes in myofibroblasts by integrating pro-fibrotic signals (*i.e.* TGFβ and Hippo/YAP1 pathways) [25] (Fig. 1 and Table 1).

Myofibroblast activation correlates with fibrosis and increased risk of cancer and many studies have described the fibroblast conversion into cancer-associated myofibroblasts as a critical event in cancer growth and progression [13].

On the other hand, healthy fibroblasts may provide tumor-suppressive signals, preventing malignant transformation in the early stage of tumor progression. Noteworthy, fibroblasts are transcriptionally dynamic and plastic and adapt their function to the evolving TME. The cross-talk with TME components, including ECM, endothelial and immune cells, by paracrine/autocrine signals can reprogram NFs to tumor-promoting phenotype, in the process of the CAF activation [70, 71].

In this context, the vital role of TFs in determining healthy tissue fibroblast specification, reprogramming and plasticity have suggested that a deeper understanding of how the TFs regulate the transition of normal fibroblasts into CAFs will provide new insight into the fibroblast differentiation and reprogramming.

In normal pancreas lineage tracing of healthy fibroblast populations suggested that the activation of a specific transcriptional program may be an early contributor to the generation of different CAF populations. Indeed, the expression of GLI1 and HOXB6 TFs associates with two different kinds of fibroblasts, with Gli1 + fibroblasts expanding dramatically and contributing to the stroma during pancreatic carcinogenesis, whereas Hoxb6 + cells do not [26]. The phenotypic conversion of NFs to CAFs has been associated with the loss of fibroblast specific signatures as revealed in lung, where the organ-specific-features of fibroblasts determined by tissue specific key TFs, including the T-box transcription factors (*i.e.* TBX2, TBX4, and TBX5), were globally downregulated in CAFs. Notably, TBX2, TBX4, and TBX5 were downregulated and hypermethylated in lung CAFs, suggesting an association between epigenetic silencing of these factors and phenotypic alteration of lung fibroblasts in cancer [27]. The study also highlighted the importance of TBX4, as involved in the super-enhancer-mediated transcriptional program in CAF activation [27] (Fig. 1 and Table 1).

## Transcriptional control of CAF generation and activation

Many studies have demonstrated that the conversion of NFs into CAFs results from the transcriptional modulation of multiple genes through different signaling pathways. The activation of CAFs is initiated by a combination of autocrine signaling, changes in ECM stiffness and composition, metabolic stress conditions, and the influence of secreted signaling molecules derived from the TME. While it is widely recognized that these transcriptional pathways are instrumental in CAF generation and activation, there is a notable gap in our understanding of the early TF changes that regulate CAF activation and phenotype stabilization. Discovering the master transcriptional mechanisms responsible for the transition from normal fibroblasts to CAFs would significantly enhance our comprehension of CAF biology and contribute to the development of more effective therapeutic strategies. In this paragraph, we thus summarize the role of specific TFs and their associated molecular pathways in contributing to NFs conversion into CAFs and in CAF activation (Fig. 1 and Table 1).

### Transcriptional repressor control of early CAF activation

The initial stages of CAF activation require intricate transcriptional and chromatin alterations. The Recombination Signal Binding Protein For Immunoglobulin Kappa J Region (CSL/RBP-Jκ) complex, a key transcriptional regulator and effector of Notch signaling and a determinant of global chromatin regulation, exerts its inherent transcriptional-repressive function to negatively regulate numerous CAF effector genes. Different TFs, including p53, activating transcription factor 3 (ATF3), and the androgen receptor transcription factor (AR), converge on CSL's transcriptional repressive functions, forming complexes that actively repress the transcription of critical CAF genes. For instance, CSL's binding to p53 serves to suppress p53 activity and override CAF senescence [28], effectively repressing stromal cell evolution and expansion. ATF3 [29] acts as a transcriptional repressor for early skin cancer-CAF activation genes, aligning with the transcriptional repressive role of CSL. ATF3 loss or down-modulation triggers CAF activation, while its overexpression exerts the opposite effect. Even at low basal levels, ATF3 binds to a significant number of genes and functions as a transcriptional repressor for early CAF activation genes. In fibroblasts of different organs, including pancreatic stellate cells, dermal and lung fibroblasts, AR and CSL jointly control key senescence and CAF effector genes. AR knockdown results in increased expression of the main CAF marker αSMA and several CAF effector genes [30].

Genome-wide analysis of AR transcription factor binding in prostate stromal fibroblasts showed that the AR binding differs between primary prostate fibroblasts and prostate cancer epithelium, suggesting that AR binding to chromatin occurs via different co-factors or other transcription factors. By applying ChIPseq and RNA sequencing of the transcriptome (RNA-Seq), the authors provided the first AR cistrome in primary prostate fetal fibroblasts and CAFs [72], strengthening the biological and clinical relevance of AR-regulated changes in prostate cancer. In prostate cancer-derived CAFs (PCa-CAFs), AR modulates the tumor-promoting abilities of CAFs, with low AR expression in CAFs associated with heightened stem cell marker gene expression in cancer epithelial cells. AR-depleted CAFs support cancer epithelial cells in forming spheroids in Matrigel and reduce IFN-γ and M-CSF-mediated promotion of stem cell marker expression in prostate cancer cells. Interestingly, AR signaling is implicated in the reduction of CCL2 and CXCL8 secretion, thus influencing prostate cancer cell migration. The study also reveals that AR occupies distinct chromatin sites in CAF-like cells as compared to prostate cancer cells, as highlighted by ChIP-seq analysis [31]. Recently, a novel role of AR loss in CAF activation has been uncovered as its loss in normal dermal fibroblasts, by either genetic or pharmacologic approaches, induces nuclear lamina A/C phosphorylation resulting in significant deformation of nuclear shape, nuclear abnormalities and ruptures during interphase of cell cycle, all features that characterize CAFs [32].

However, these negative regulators may adopt different roles in the late stages of tumor progression by influencing paracrine CAF-cancer cell interactions, suggesting that the evaluation of determinants of CAF functionality has to be considered in the context of the TME evolution during cancer progression. Indeed, in the later stages of CAF activation, with the alteration of p53-transcriptional targets, p53 switches into a positive regulator, mediating CAF pro-tumoral functions [54]. A similar effect occurs for the ectopic overexpression of ATF3 that, in several types of cancer, promotes CAF proliferation and in parallel the growth of adjacent cancer cell lines in a non-cell autonomous manner [55].

### TFs in the regulation of autocrine transcriptional program

CAF behavior is profoundly influenced by various major signaling pathways, including TGF-β, Hedgehog, Notch, Wnt, Hippo, NF-κB, JAK/STAT, MAPK, and PI3K/AKT pathways. While the detailed coverage of all these signaling pathways involved in CAF activation is extensive and beyond the scope of this review, we highlight that the CAF autocrine signals exert a central role on specific TFs that, once activated, plays, in turn, a pivotal role in CAF differentiation, activation, and pro-tumorigenic functions. Notably, the activation of SMAD2/3 represents a critical element in the regulation of TGF-β and SDF-1 autocrine signaling, which is essential for myofibroblast differentiation, phenotype maintenance, and associated tumor-promoting activities [33]. When SMAD2/3 is phosphorylated, it forms a heterotrimeric complex with SMAD4 and translocates into the nucleus, where it acts as a transcription factor complex responsible for transcribing TGF-β target genes, including α-SMA.

The activation of Heat-Shock Factor 1 (HSF1) in breast cancer CAFs supports the expression of pro-tumoral gene expression programs mediated by TGF-β/SDF1 in both fibroblasts and cancer cells [34]. Another illustrative example is furnished by the interaction between RUNX3 and the proto-oncogene MYC, and the consecutive binding to the promoter of TGF-β1, leading to CAF activation and tumor progression in colorectal cancer [35]. These findings strengthen the concept that once fibroblasts become activated, they may maintain their CAF status in a cell-autonomous way, underlying the importance to decipher the responsible mechanisms and molecules for designing CAF-specific therapeutic approaches. To this regard, our group has recently found that the hMENA protein, with a crucial role in CAF-cancer cell pro-tumoral cross-talk [73], sustains autocrine TGF-β1 signaling contributing to CAF activation and paracrine TGF-β1 signaling, also inducing SMAD2/3 and Signal transducer and activator of transcription 1 (STAT1) activation in tumor cells (unpublished data).

### Mechanosensitive transcriptional signaling

The dynamic interplay among fibroblasts, the ECM, and environmental cues, particularly mechanotransduction, constitutes a central regulator of the transition from NFs to CAFs. Mechanical and biochemical signals from ECM are conveyed into cells by integrins, mainly by the β1 integrin subfamily. Once the integrins are activated, the RhoA/ROCK enhances collagen and fibronectin accumulation. The assembly of F-actin is then facilitated by Talin/FAK to promote signal transduction. The connection of actin with myosin II conveys the mechanical cues to the nucleus [74].

As primary architects of ECM synthesis and remodeling, CAFs exert considerable influence over ECM stiffness. This alteration promotes focal adhesion assembly and enhances cytoskeletal tension, thereby amplifying growth factor receptor signaling-dependent activation in tumor cells.

Of note, TFs play a pivotal role in translating mechanical cues generated by the matrix stiffness into biochemical signaling, mainly with two key transcriptional regulatory networks: YAP-TEAD (Yes-Associated Protein-Transcriptional Enhanced Associate Domain) and MRTF-SRF (Myocardin-Related Transcription Factor-Serum Response Factor). These networks are essential regulators of CAF activation and function in response to extracellular signals and mechanical stimuli. Both YAP and MRTF transcriptional pathways are downstream of RhoA/ROCK-mediated actin cytoskeletal rearrangements, and their crosstalk involves various proteins involved in actin dynamic regulation [38]. The prominent role of actin cytoskeletal changes and actin regulatory proteins in communicating extracellular stimuli to the nucleus and controlling gene expression has been demonstrated [75]. To this regard, our group has reported that the actin regulatory protein hMENA sustains the expression of the ECM receptor β1 integrin by affecting the SRF cofactor MRTF-A nuclear translocation, and SRF activity [76]. The role of MENA at the nucleus has been described in a recent paper reporting that MENA links the adhesome, cytoarchitecture and gene activity, by controlling at the nuclear membrane the LINC (Linkers of the nucleoskeleton and cytoskeleton) complex, chromatin organization and cancer specific immune genes [77].

When YAP is translocated to the nucleus with a subsequent activation of YAP/TAZ target genes, triggered by mechanical stimuli like matrix stiffness, cytoskeletal tension, nuclear deformation, and extracellular mechanical tension, many pro-tumorigenic CAF functions are activated [36]. Several mechanisms in regulating the nuclear translocation and activation of YAP in CAFs have been proposed. HSF1 positively regulates this process by activating Dickkopf-3 (DKK3), leading to the simultaneous activation of β-catenin and YAP/TAZ, required for the induction of CAF pro-tumoral phenotype [37]. On the other hand, the scaffolding protein Cerebral Cavernous Malformations 3 (CCM3) acts as a negative regulator of YAP/TAZ signaling in fibroblasts, functioning as a gatekeeper in focal adhesions and mechanotransduction [78].

The crosstalk between YAP-TEAD and MRTF-SRF pathways is crucial in maintaining the CAF activated state, suggesting a synergy in potentiating TGFβ signaling by YAP to elevate MRTF-SRF activity, which, in turn, influences YAP-TEAD signaling through their ability to affect actin cytoskeletal dynamics [38].

A crosstalk between TFs in a stiff tumor microenvironment has been also reported for the epithelial-mesenchymal transition transcription factors (EMT-TFs) SNAIL1 and YAP, where SNAIL1 acts as a mechano-responsive transcriptional regulator in CAFs, enhancing YAP activity. Its activation through ROCK- and ERK2-dependent pathways controls the fibrogenic response of CAFs and increases YAP activity triggered by matrix stiffness in CAFs [39]. YAP/TEAD/SLUG association has been shown to mediate the resistance to combined EGFR/MEK inhibition by inducing dormancy in non-small-cell lung cancer cells, through the direct inhibition of the pro-apoptotic protein BMF (Bcl2 Modifying Factor) [79].

An additional transcriptional regulatory mechanism of mechanosensing is mediated in CAFs by the Zinc Finger transcription factor Protein ZNF416. By analyzing the effect of matrix stiffness on genome-wide chromatin accessibility in freshly isolated lung fibroblasts using ATAC-seq, the authors identified ZNF416 as a critical transcriptional regulator of fibroblast contractile activation, proliferation and matrix synthesis [40].

All these data highlight the relevance to identify key TFs crucial in translating mechanical cues as a potentially targetable mechanism to inhibit CAF activation and modify TME stiffness.

### TFs in metabolic reprogramming of CAFs

To support their energy demand, highly proliferative cancer cells enhance their ability to uptake nutrients, such as glucose and glutamine and the generation of metabolic byproducts including lactic acid and ammonium (NH4 +) induces a metabolic reprogramming. Interestingly, the metabolic reprogramming also plays a pivotal role in shaping the distinct behavior of CAFs to fuel neighboring tumor cells [80].

One central aspect of metabolic reprogramming in tumors involves glycolytic changes induced by hypoxia and hypoxia-inducible factor 1 alpha (HIF-1α) [81, 82]. HIF-1α directly stimulates the transcription of glycolytic enzymes [83] and chronic hypoxia reprograms normal fibroblasts into CAFs characterized by higher levels of both HIF-1α transcripts and proteins. These CAFs also exhibit a pro-glycolytic transcriptome and metabolome, actively fueling the metabolism of breast cancer cells and promoting tumor growth [41].

In this context, the pituitary-specific POU homeodomain transcription factor (POU1F1) has emerged as a critical regulator in the metabolic reprogramming of human breast tumor cells, modifying the phenotype of both cancer cells and fibroblasts to promote cancer progression [42]. Mechanistically, POU1F1 transcriptionally regulates the lactate dehydrogenase A (LDHA) gene, and the resulting lactate production induces the activation of NFs into CAFs. Notably, POU1F1 knockdown or LDHA blockade partially reverse CAF activation.

Similarly, the activation of AP-1 transcription factors, such as c-FOS and c-JUN, has been found to modulate the expression of glycolytic enzymes required for CAF activation [43].

The shift in cancer metabolism towards aerobic glycolysis is closely linked to mitochondrial dysfunction. The expression of the mitochondrial transcription factor A (TFAM) in CAFs has been revealed as a critical regulator of CAF phenotype. Studies have shown that downregulation of TFAM in fibroblasts results in the loss of the Caveolin 1 protein expression, a potent stromal biomarker for tumor progression. Furthermore, TFAM knockdown promotes tumor formation in an MDA-MB-231 xenograft model in mice [44].

### EMT-related transcription factors

EMT-TFs have emerged as significant players in the regulation of CAFs, with their effects extending to tumor progression and therapy response [47]. These EMT-TFs, mainly including SNAILl, Twist-related protein 1 (TWIST1), ZEB1 and ZEB2, are pivotal in activating CAFs in different cancer contexts [84–86]. They are also instrumental in creating paracrine stimuli that profoundly influence adjacent cancer cells, significantly affecting tumor progression and therapeutic responses [85, 87].

SNAIL1, in particular, is considered a marker of activated fibroblasts within the tumor stroma [88]. In pancreatic cancer, SNAIL1 is predominantly observed in the nuclei of stromal cells, with limited presence in cancer cells. SNAIL1 activity is widely recognized as a requirement for the pro-tumoral activity of CAFs across various cancers. A recent research has revealed an intriguing role for stromal SNAIL1 in melanoma biology [48] and demonstrated that stromal SNAIL1 expression directly regulates fibroblast activation protein alpha (FAP) transcription in fibroblasts, and induces melanoma growth by promoting an immunosuppressive microenvironment and a decrease in anti-tumour immunity. Notably SNAIL1 induces the expression of fibronectin and collagen while promoting matrix rigidity through the regulation of the collagen-crosslinking enzyme LOX1, as discussed above [39].

Another pivotal EMT-TF, TWIST, plays a significant role in fibroblast activation in multiple studies [86, 89], and acts as a key component in a complex circuit comprising TWIST1, Paired-related homeobox 1 (PRRX1), and Tenascin-C (TNC). This circuit functions as an "ON/OFF switch" for fibroblast activation, with implications for pathologic conditions like wound healing and fibrotic diseases [49]. PRRX1 itself plays a critical role in tuning CAF activation as evidenced in PDAC PRRX1 knock-out mouse model [50].

Furthermore, ZEB1 has been identified as a critical determinant of pro-tumoral CAF features, and its role as a key regulator of CAF plasticity and heterogeneity is detailed in paragraph 3.2 [51].

The involvement of EMT-TFs in CAF activation underscores the significant impact that master TFs may exert on CAF functionality and on the complexity of the TME and its mesenchymal traits which have been suggested crucial in therapy response [90, 91].

## Transcriptional control of CAF heterogeneity and plasticity

**Fig. 2:**
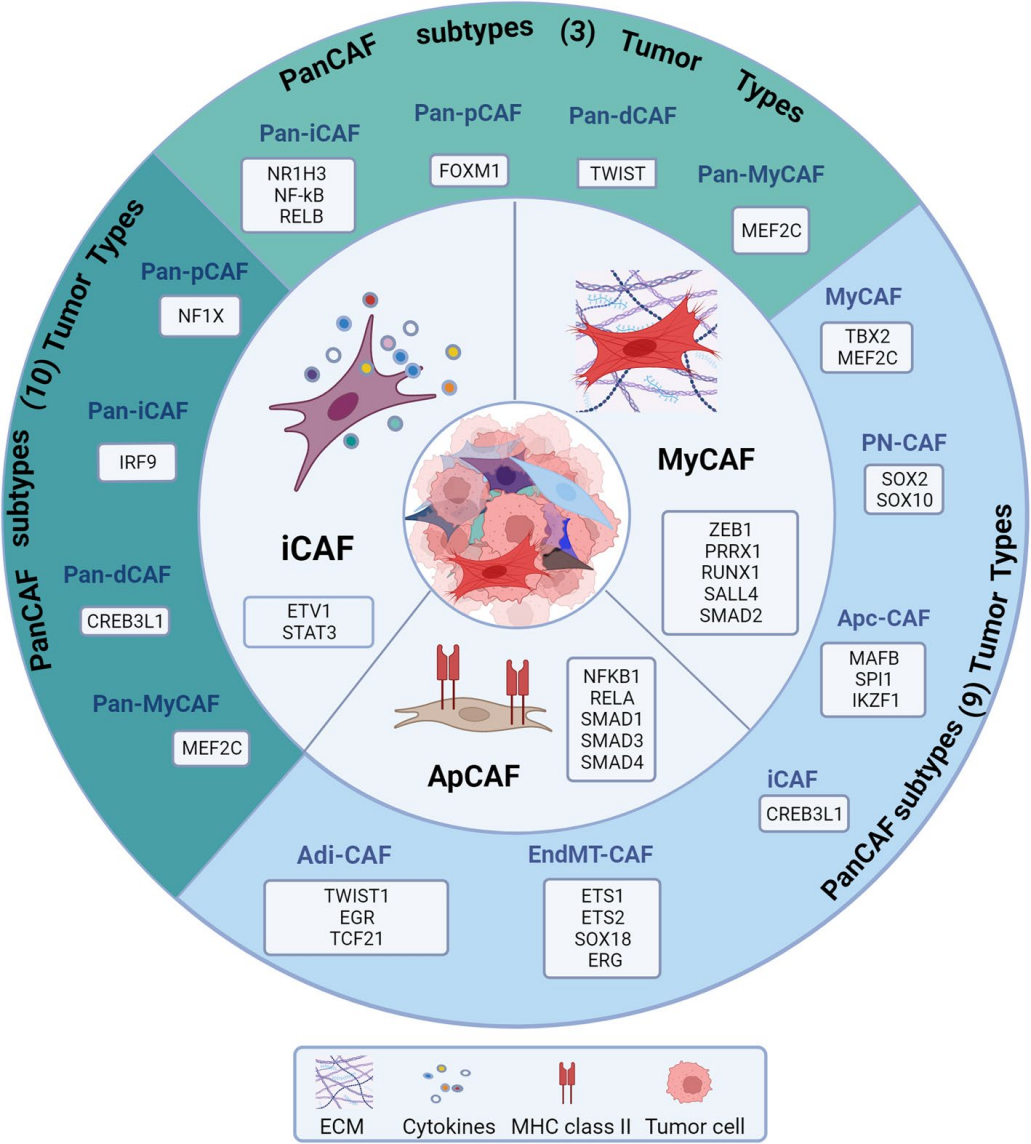
Transcription factors specifying CAF Subtypes. Three main CAF populations have been described: inflammatory, iCAF characterized by cytokine/chemokine secretion; myofibroblasts, myCAF providing ECM modulation, collagen deposition; antigen-presenting CAF, apCAF expressing major histocompatibility complex (MHC) class II genes. TFs associated with each of the CAF subtype are reported. Additional subtypes were defined by scRNA-seq. From 3 tumor histotypes, melanoma, head and neck squamous cell carcinoma, and lung cancer, 6 different subtypes including pan-myCAF, pan-dCAF, pan-iCAF, have been identified and associated with specific transcription factors (3 Tumor types) [8]. From scRNA-seq data of 9 studies of pan-cancer CAF atlas, four different CAF subtypes, namely progenitor CAF (proCAF), inflammatory CAF (iCAF), myofibroblastic CAF (myCAF), and matrix-producing CAF (matCAF) emerged as associated with core regulatory network of transcription factors (TFs), that are highly activated in CAF subtypes with similar functionality (9 tumor types) [92]. Finally, transcriptomic profiles of fibroblasts from multiple tumor types (10 tumor types) different clusters have been identified, with three major components, including cancer-associated myofibroblasts (CAFmyo), inflammatory CAFs (CAFinfla), and adipogenic CAFs (CAFadi), along with minor components, such as endothelial-to-mesenchymal transition CAF (CAFEndMT), peripheral nerve-like CAF (CAFPN), and antigen-presenting CAF (CAFap) [9]. Created with BioRender.com

This section explores the role of specific TFs and their related molecular pathways in contributing to the definition of CAF subset specification, as detailed in Fig. 2 and Table 1.

### CAF subtypes

Recently, scRNA-seq and other approaches have revealed transcriptionally and functionally distinct CAF subpopulations, myofibroblastic CAFs (myCAF), inflammatory CAFs (iCAF), and antigen presenting CAFs (apCAF) (Fig. 2, core). MyCAF characterized by the ECM modulation, collagen deposition, contraction and adhesion activities and iCAF characterized by cytokine/chemokine secretion and crosstalk with immune cells, have been identified in all organs and across different cancer types [93, 94]. Unlike iCAF and myCAF subtypes, the apCAF subtype expressing major histocompatibility complex (MHC) class II genes has so far been described only in specific tumors including pancreatic ductal adenocarcinoma (PDAC) [95], where they contribute to tumor immune regulation [96].

Besides these main CAF subsets, an increasing number of other and organ-specific CAF subsets are continuously identified by scRNA-seq analyses. Different studies on a range of tumors have defined additional CAF subtypes associated with specific transcription factors (Fig. 2, outer layer), whose roles in CAF subtype specificity are discussed in the following sections.

Looking at three tumor histotypes, melanoma, head and neck squamous cell carcinoma, and lung cancer, six different subtypes, including pan-myCAF and pan-iCAF, have been identified [8], whereas a very recent scRNA-seq dataset of human breast cancer has defined nine CAF and one pericyte populations, generalized across several cancer types, providing a useful list of marker genes for general identification and functional interpretation of different CAF types [97].

In NSCLC, three functional subtypes of CAFs have been identified, Subtype I, II and III CAFs. The former two factors with high levels of hepatocyte growth factor (HGF) and fibroblast Growth Factor 7 (FGF7) expression are associated with unfavorable response to targeted therapies. Subtype III CAFs with high p-SMAD2 expression is associated with better clinical response and immune cell migration [65].

In breast cancer, Y. Kieffer and coauthors [98] identified 8 different CAF clusters with 3 clusters belonging to iCAF, 5 clusters belonging to myCAF which included the ecm-myCAF subset, highly enriched in ECM protein expression and immunosuppressive function. In breast cancer four more CAF subtypes have been identified, namely vascular CAFs (vCAFs), matrix CAFs (mCAFs), cycling CAFs (cCAFs), and developmental CAFs (dCAFs), mainly with overlapping function with the myCAF subtype described in pancreatic tumors [94].

In a single-cell transcriptomic study aimed to characterize the kinetics of increasing functional divergence and heterogeneity during tumor development, three different melanoma stromal populations, referred to as Stromal 1, 2, and 3 were identified. These were defined by specific CAF marker combinations and displayed distinct functional and temporal features [99].

Overall, these studies based on single-cell sequencing technologies have undoubtedly rapidly evolved the understanding of CAF heterogeneity, however a unified functional classification of different CAF subtypes is still lacking. Moving toward this direction, the identification of master TF and specific signaling pathway activation rather than single gene/protein expression would represent a valid approach to capture the identities and states of CAF subtypes [100, 101].

### Transcriptional control of CAF heterogeneity and plasticity

Recent studies that have examined the phenotypic and functional heterogeneity of CAFs at the single-cell RNA level, have demonstrated the enrichment of gene regulatory networks involving TFs in various CAF subcategories found in different types of tumors [8, 9, 92]. This supports the notion that TFs play a central role in shaping the distinctive functional subtypes of CAFs. Moreover, TFs also have a critical role in the functional molecular co-evolution of metastasis-associated fibroblasts, with the example of MYC playing a crucial role in rewiring metastasis-associated fibroblasts in lung cancer. These findings emphasize the significance of TFs in the regulation and functional diversity of CAFs [59].

ZEB1, a paradigmatic EMT-related TF, has emerged as a key regulator in CAF plasticity. Using a mouse model for colitis-associated cancer and sporadic metastatic colorectal cancer (CRC), ZEB1 was found to balance myofibroblastic and inflammatory functions in colorectal cancer (CRC) CAFs [51]. Indeed, ZEB1 deletion in fibroblasts promoted inflammation-driven cancer initiation and progression in a context- and stage disease-dependent manner. Remarkably, ZEB1-deficient CAFs exhibited an enhanced inflammatory profile and reduced myCAF features, as revealed by scRNA-seq. This analysis unveiled an increased expression of inflammatory genes (*Tnfaip3, Cxcl1, Icam1, Ccrl2, Nfkbia, Il1b,* and *Irf1*) alongside a decreased expression of genes associated with extracellular matrix organization. ZEB1 loss in CAFs resulted in reduced collagen deposition while amplifying inflammatory signaling and immune cell attraction. Intriguingly, specific depletion of ZEB1 in CAFs heightened sensitivity to immune checkpoint inhibition, suggesting ZEB1 as a therapeutic target in addition to its role as a prognostic biomarker (Fig. 2 and Table 1).

Also the TF PRRX1 has a role in defining functional CAF subsets, as it has been recognized as a master regulator of myofibroblast-like functions in CAFs [61]. Extensive in vivo and in vitro studies, including large-scale ChIP-seq, RNAseq, and scRNA-seq, have revealed PRRX1's role as a lineage-specific TF of a subset of myCAFs, linked to cancer promotion and progression. Moreover, PRRX1-mediated CAF plasticity has been shown to significantly impact PDAC biology and therapeutic resistance. PRRX1 itself plays a critical role in tuning CAF activation, enabling a dynamic transition between a dormant and an activated state, as demonstrated by the use of a PDAC PRRX1 knock-out mouse model. The study showed the generation of highly activated CAFs with increased ECM deposition. This led to improved tumor differentiation, heightened sensitivity to chemotherapy, and disruption of systemic tumor dissemination [50] (Fig. 2 and Table 1).

Mainly associated with EMT and extracellular matrix-related processes, RUNX1 and 2 appear as vital regulators of CAF functionality. RUNX1 has been associated with the maintenance of the proliferative status of TGF-β-activated mesenchymal stem cell progenitors in normal prostate [45]. During prostate cancer stroma formation, it fosters the differentiation of mesenchymal stem cells into myCAFs, affecting TGF-β1-stimulated gene expression (Fig. 2 and Table 1).

RUNX2 has also garnered attention as a CAF-related transcription factor with prognostic value. Indeed, it is abnormally expressed in bladder cancer cells (BLCA), and its inhibition in CAFs reduces the migratory, invasive, and proliferative capabilities of BLCA cells [52]. Notably, in patient-derived skin squamous cell carcinoma (SCC) CAFs, RUNX2 has been linked to a peculiar CAF subtype, influencing collagen-related gene expression and tumorigenic matrix production [57]. Studying the molecular parallels between early wound fibroblasts and CAFs, the authors identified three main wound-associated CAF subtypes: contractile, collagen-forming, and elastin-forming CAFs by analyzing bulk and single-cell sequenced wound fibroblasts and CAFs. Of note, they found that the RUNX2 TF identifies an "early wound" CAF subtype, localized to the inner tumor stroma and expressing collagen-related genes. The transient knock-down of RUNX2 in patient-derived skin SCC CAFs reduced the expression of CAF markers associated with the production of a tumorigenic matrix and reduced the cancer cell growth on decellularized ECM deposited by CAFs (Fig. 1 and Table 1).

The fundamental role of RUNX1 and 2 in the regulation of gene activity in CAF specification is greatly strengthened in a recent study [58]. With the aim of identifying regulatory elements and TFs associated with the different stages of transformation from normal colon to carcinoma, the Authors identified RUNX11 as strongly associated (more than RUNX2) with widespread chromatin accessibility and gene activity in CAFs. Notably, trajectory analysis of chromatin accessibility profiling at the single cell level of normal fibroblasts, preCAF and CAF clusters showed increased activity of FOX family transcription factors in the intermediate states, which is followed by increased activity of RUNX1 JUN, FOS, and CEBP motifs in CAFs. This putative trajectory of TF accessibility and expression changes in parallel with the development of CAFs from normal colonic fibroblasts through pre-CAF transformation [58] (Table 1).

A role in CAF subset specification has been found for Spalt-like Transcription Factor 4 (SALL4), mainly known as directly involved in the maintaining of the pluripotency and self-renewal functions of embryonic and hematopoietic stem cells [102, 103]. SALL4 transcriptional activity has been linked to a specific stromal signature in TGF-β-activated CAF subsets associated with invasiveness and poorer clinical outcomes in PDAC.

[62]. scRNA-seq analysis of PDAC cells sorted from patients revealed that SALL4 transcripts are limited to a specific CAF subset characterized by high levels of Leucine Rich Repeat Containing 15 (LRRC15), a marker of TGF-β-activated fibroblasts, in the TGF-β1 driven CAF cluster and identified a crucial crosstalk between TGF-β1 and SALL4 in promoting CAF subpopulation heterogeneity and stromal functions along PDAC oncogenesis (Fig. 2 and Table 1). The gene expression profile associated with the SALL4-related network mirrors that myCAF subsets, previously associated with triple-negative breast cancer (CAF-S1) by Kieffer et al., reinforcing the significance of SALL4 in the stromal definition. Moreover, the SALL4 high fibroblast subset is characterized by the TGF-β1-mediated overexpression of stemness-related transcription factors, such as SRY-Box Transcription Factor 2 (SOX2), octamer-binding transcription factor (OCT3/4), and NANOG, likely linked to stromal cell renewal [98].

That stem cell transcriptional signature activation may represent an important event in CAF activation is sustained by literature data [63] which described an interesting molecular mechanism whereby the up-regulation of the TF SOX2, a known master regulator of lineage cell plasticity [104], leads to the reprogramming of colonic fibroblasts to support the tumor progression of human CMS4, the most aggressive colorectal cancer subtype. The authors demonstrated that the TGF-β1-mediated loss of PKCζ (a member of the atypical protein kinase C (aPKC) family) in the stroma of CMS4 tumors promoted a SOX2-dependent fibroblast switch, leading to the generation of a SFRP1/2 (Secreted Frizzled-related protein-1/2)-expressing CAF population, namely Cluster 0. Notably, this switch, by impacting the immune system, contributes to immunosurveillance impairment and to the development of an immunosuppressive TME [63] (Table 1).

### TFs in shaping different CAF population induced by distinct signaling pathways

Heterogeneity of CAFs also results from the activation of distinct signaling pathways, controlled by specific TFs which may determine the balance between signals with opposite functions. As an example, ETS1 Variant Transcription Factor (ETV1) controls the duality of FGF/TGF-β signaling in shaping two different CAF populations in non-desmoplastic skin squamous cell carcinomas (SCC) [64]. TGF-β1, while inducing the expression of multiple ECM proteins, growth factors, and markers of CAF activation such as actin alpha 2, smooth Muscle (ACTA2) and integrin subunit alpha 11 (ITGA11), reduces the expression of FGF2 regulated genes. On the contrary, FGF2 induces the expression of growth factors, and inflammatory cytokines and inhibits TGF-β1-mediated gene regulation in CAFs. A global gene expression analysis (GSEA) has identified ETV1 as a critical determinant of this FGF-TGF-β dualism in CAF activation. The up-regulation of ETV1 is sufficient to induce the expression of FGF-controlled genes while its silencing in CAFs suppresses these genes and induces those under positive TGF-β control. Thus, ETV1 mediated activity leads to the generation of two distinct CAF populations that converge on promoting cancer development, through the activation of two different processes: EMT (in primary human dermal fibroblasts with increased TGF-β signaling) *vs* macrophage infiltration (in fibroblasts with increased FGF signaling). Notably, TGF-β treatment confers the myCAF trait to human dermal fibroblasts, while exposure to FGF2 induces the iCAF trait via activation of an ETV1 transcription factor in these fibroblasts) [64] (Fig. 2, core and Table 1). The contribution of ETV1 to fibroblast functional heterogeneity has also been clearly demonstrated in NSCLC. By establishing a living biobank of CAFs derived from biopsies of NSCLC patients [65] harboring specific oncogenic alterations, the authors identified, as previously mentioned, three additional functional CAF subtypes (subtypes I, II and III) based on their level of HGF and FGF7 expression and the ability to overcome the CAF-mediated tyrosine kinase inhibitors (TKI) resistance. In particular, subtype I and II CAFs have high HGF and FGF7 expression and protect cancer cells while subtype III CAFs are associated with better clinical response and immune cell migration in the tumor. All functional CAF differences are governed by CAF intrinsic TGF-β signaling which suppresses HGF and FGF7 expression. Notably, the Authors found that this CAF intrinsic TGF-β signaling and the derived fibroblast functional heterogeneity is governed by specific transcriptional networks which include ETV1 and TBX2 TFs. Both ETV1 and TBX2 were instead downregulated in CAFs exposed to TGF-β and ETV1 knocking-down reduced the HGF expression in subtypes I and II, whereas its overexpression enhanced HGF expression in subtype III (Fig. 2, core and Table 1).

Interestingly, the CAF-mediated TKI resistance was reduced but not fully abolished by ETV1/TBX2 knockdown, indicating that additional transcription factors are likely required for this CAF-related function.

Another example of CAF plasticity determined by the activation of distinct signaling pathways has been recently evidenced in pancreatic cancer progression, where IL-1 and TGF-β signaling induce the up-regulation of NF-kB and SMADs TFs during mesothelial cells to apCAFs transition [96].

### Role of TFs in dictating CAF heterogeneity mediated by cancer cell contextual cues

The control of CAF heterogeneity and plasticity is strongly influenced by different cancer cell-derived signals such as TGF-β, Wnt, SDF-1, IL-6, and IL-1α [105]. This was first clearly demonstrated in PDAC, where tumor-derived TGF-β and IL-1 ligand secretion promote the induction of myCAF and iCAF, respectively. Tumor-secreted IL-1 induces Leukemia inhibitory factor (LIF) expression and downstream JAK/STAT3 activation, generating inflammatory CAFs. In contrast, TGF-β antagonizes this process by downregulating IL1R1 expression in CAFs and promoting differentiation into myCAFs [66]. Notably, the specific knockout of STAT3 (but not STAT1) in naïve pancreatic stellate PDAC cells (PSCs) reduced the expression of iCAF marker genes, emphasizing STAT3's role in regulating iCAF genes. The authors confirmed the role of STAT3 in regulating iCAF marker genes by conducting a DNA motif analysis on the promoters of genes that were differentially expressed between myCAFs and iCAFs, as previously identified in the RNA-sequencing dataset [67]. Indeed, STAT3 motifs were found to be enriched in the promoters of several iCAF genes, including IL6. This observation highlights the significance of STAT-3 expression in CAFs which appears to be essential also for the CAF-mediated pro-tumorigenic functions, as demonstrated in breast cancer through a STAT3-driven secretion of soluble mediators [56]. This suggests that STAT-3 plays a pivotal role in defining a specific CAF subtype and in sustaining the pro-tumorigenic functions of CAFs, as illustrated in Fig. 2 and Table 1 and discussed in paragraph 4.2.

Furthermore, the transcription factor myeloid zinc finger 1 (MZF1) has been shown to mediate tumor cell-induced transformation of mesenchymal stem cells (MSCs) into CAFs. This transformation occurs through the regulation of the osteopontin (OPN)-TGF-β1 pathway. MZF1 influences the tumor-derived MSC-to-myCAF transformation induced by OPN-mediated TGF-β1 production in MSCs, which then adopt a myCAF phenotype. This process also requires the expression of cancer stemness TFs OCT-4, NANOG, and SOX2, emphasizing the role of TFs in cancer cell-myCAF crosstalk [68], as also demonstrated in MBA-MB-231 breast cancer and HepG2 hepatocellular carcinoma cells [106]. In addition, MZF1 phosphorylation in mesenchymal stem cells drives the osteopontin-mediated CAF phenotype, which then increases the cancer cell stemness profile [107] (Table 1).

Additionally, early changes in TF activity were observed in indirect co-cultures of normal prostate fibroblasts with normal or cancerous epithelial prostate cells. The activation of different TFs was identified depending on whether fibroblasts were co-cultured with normal (RNF4, SNAPC1) or cancerous epithelial cells (GTF3C1, and THRAP3). The pathway analysis of these differentially activated transcription factors revealed the involvement of two main pathways associated with CAF transformation, PTEN and RUNX1 associated transcription [53].

The transcription factor ZEB1 plays a fundamental role in CAF heterogeneity induced by cancer cell contextual cues, particularly in lung adenocarcinoma (LUAD) where ZEB1-overexpressing tumor cells can reprogram CAFs through a ZEB1-dependent secretory program. This leads to the directed movement of CAFs towards invasive projections due to a ZEB1-driven CAF repulsion process. ZEB1-driven EMT, in turn, sensitizes LUAD cells to pro-metastatic signals from CAFs. Experimental findings have demonstrated that CAFs lose their ability to enhance metastatic activity of tumor cells when depleted of ZEB1 [60] (Table 1).

A different mechanism of CAF transformation involves the zinc finger protein 32 transcription factor (ZNF32). When ZNF32 is knocked down in MCF7 cells, the transformation of NFs into CAFs is facilitated, while its overexpression in MDA-MB-231 cells achieves the opposite effect. ZNF32 inhibits TGF-β1 transcription in breast cancer cells by directly binding to the TGF-β1 promoter, preventing fibroblast activation [46] (Table 1).

Lastly, the p53 mutational status of pancreatic cancer cells can educate CAFs to establish a pro-metastatic and chemoresistant TME [69] (Table 1).

### Segregation of specific TFs in CAF subtypes

Recent advances in single-cell RNA analysis have yielded further evidence regarding the segregation of specific TFs in different CAF subtypes (as depicted in Fig. 2, outer layer). Six pan-CAF subtypes, which encompass pan-myCAF (myofibroblast-like CAFs), pan-dCAF (desmoplastic CAFs), pan-iCAF, and pan-iCAF-2 (inflammatory-like CAFs), pan-nCAF (normal myofibroblasts), and pan-pCAF (proliferating CAFs), have been identified in melanoma, head and neck squamous cell carcinoma, and lung cancer [8].

These subtypes are characterized by distinct TFs and regulatory gene programs. Myocyte Enhancer Factor 2 C (MEF2C) expression, along with its target genes ACTA2 and myosin light chain kinase (MYLK), was enriched in pan-myCAF. In contrast, pan-dCAFs were marked by the presence of TWIST1, and its associated target genes (TWIST2, COL1A1, MMP2). Pan-iCAFs and pan-iCAFs-2 were distinguished by the high levels of the inflammation-associated TF Nuclear Receptor Subfamily 1 Group H Member 3 (NR1H3) and its target genes (IL33, CXCL14, CXCL12), as well as the high expression of the NFκB subunit RELB, a cofactor involved in promoting inflammatory transcriptional programs. Pan-pCAFs, associated with cell proliferation, exhibited FOXM1 expression and its target genes (BIRC5, CDK1), providing insights into the gene regulatory networks underlying CAF heterogeneity. The pivotal role of TFs in determining CAF subtypes and their functional roles is further supported by recent data in the literature. A study of single-cell gene expression including 407 samples from nine cancer types, has led to the development of a pan-cancer CAF atlas. This atlas identified four different CAF subtypes, namely progenitor CAF (proCAF), inflammatory CAF (iCAF), myofibroblastic CAF (myCAF), and matrix-producing CAF (matCAF). A core regulatory network of TFs that are highly activated in CAF subtypes with similar functionality was unveiled. The top 100 enriched TFs for each CAF subtype across different cancer types were analyzed, revealing TFs common to all cancer types. TFs regulating cell proliferation, such as Nuclear factor 1 X-type (NFIX), were predominantly expressed in the proCAF subtype. Interferon regulatory factor 9 (IRF9), a TF governing immune response, was found to be predominantly expressed in iCAF, while MEF2C was overwhelmingly expressed in myCAF [92]. Conversely, the transcription factor cAMP response element–binding protein 3–like 1 (CREB3L1), which regulates collagen formation, was found to be exclusively expressed in matCAF. These active TF regulatory networks, characterizing the various CAF subtypes, align with their distinct functional roles.

Notably, MEF2C, a lineage-specific transcription factor that primarily regulates myogenesis and angiogenesis, was overwhelmingly expressed in the myCAF subtype (Fig. 2, outer layer). A recent machine-learning-based approach, integrating bulk and scRNA-seq to decipher the communication network between CAFs and tumor cells, has unveiled the specific activation of the MEF2C regulon in CAFs. The target genes of the MEF2 regulon identified in this study constitute a cell communication gene signature that is effective in predicting the prognosis and response to immune checkpoint inhibitor therapy of clear cell renal cell carcinoma (ccRCC) patients [108].

Furthermore, recent investigations have delved into single-cell profiles across multiple cancer types, leading to the identification of different clusters. Notably, a clear separation was observed between NFs and other subtypes. Among the overall population of CAFs, three predominant components were identified: cancer-associated myofibroblasts (CAFmyo), inflammatory CAFs (CAFinfla), and adipogenic CAFs (CAFadi), alongside minor components like endothelial-to-mesenchymal transition CAF (CAFEndMT), peripheral nerve-like CAF (CAFPN), and antigen-presenting CAF (CAFap) [9].

The analysis of gene regulatory networks through single-cell regulatory network inference and clustering has revealed the activation of specific regulons in distinct CAF subtypes [109]. This highlights that each subtype differentiates from others due to the activation of specific trajectories of gene expression. Interestingly, when CAFs from various cancer types were combined in an attempt to define a general activation process of CAFs, three distinct states were identified. CAFstate1 was marked by the dominance of NFs, while CAFstate2 was characterized by a prevalence of CAFmyo, and CAF state3 showed a dominance of CAFadi/CAFinfla. Importantly, the regulatory activity and expression of CREB3L1, recently identified as a key regulator of anaplastic thyroid cancer (ATC) progression, [110], gradually increased along the activation trajectory of CAFs, irrespective of tissue type, underscoring its pivotal role in the general dedifferentiation process during CAF activation, which is shared across different cancer types.

## TFs in CAFs as clinical potential therapeutic targets

Therapies targeting relevant signaling pathways involved in CAF generation, activation and in pro-tumoral phenotype of CAFs [111], although promising efficacy in preclinical and clinical trials, are not totally effective, probably due to the activation of compensatory signaling. Furthermore, multiple attempts to target them also in combination with other therapeutic approaches, *i.e. *chemotherapy or immunotherapy, have been largely unsuccessful in the clinic. Different reasons account for the clinical failure of CAF targeting strategies, but the major reason is the lack of fundamental knowledge on the origin, functional heterogeneity and plasticity of CAFs.

Transcription factors, as downstream effectors of many signaling pathways, represent attractive drug targets. Targeting TFs might be more specific and might induce fewer side effects, than targeting the upstream signaling pathways. TFs have been historically considered “undruggable”, however, recent advances in technologies shed new light on the TF-targeting strategies, as a part of targeted drug discovery and a number of TF specific drugs are in clinical trials for cancer diseases [112–114].

These new approaches may include both direct and indirect strategies in targeting TFs such as: 1) the control of the TF gene expression, 2) the inhibition of protein-protein interactions between TF and their binding partners, 3) the prevention of the functional association of TFs with co-factors, and 4) the selective TF protein degradation. In particular, the last represents a rapidly exploding drug discovery strategy that mainly uses two classes of small molecules, the proteolysis targeting chimeras (PROTACs) or molecular glues, which mediate TF targeted protein degradation via the ubiquitin - proteasome pathway through distinct mechanisms. Several PROTACs-based TF-targeting platforms have been developed as a tool to advance drugging TFs and a number of PROTACs targeting TFs, including STAT3, are currently undergoing clinical trials [115].

Recently, among the small molecule degraders emerging as a promising therapeutic approach, cereblon E3 ligase modulators (CELMoDs) have been developed and are currently being tested in clinical trials as monotherapy (NCT02848001) or in combination (NCT04336982) for acute myeloid leukemia and/or myelodysplastic syndrome [116].

Novel approaches, currently being tested, could substantially improve the ability to modulate this important class of proteins. A new way to selectively inhibit the p53 activity by controlling its binding to Mediator, a complex containing 26 subunits required for RNA polymerase II (pol II) transcription, has been proposed [117]. This novel method may potentially target other TFs by targeting distinct Mediator subunits, leading to the selective alteration of gene expression patterns.

### Clinical potential of TF targeting for the inhibition of CAF activation/functions and cancer cell/CAF crosstalk

## Targeting STAT3

**Fig. 3:**
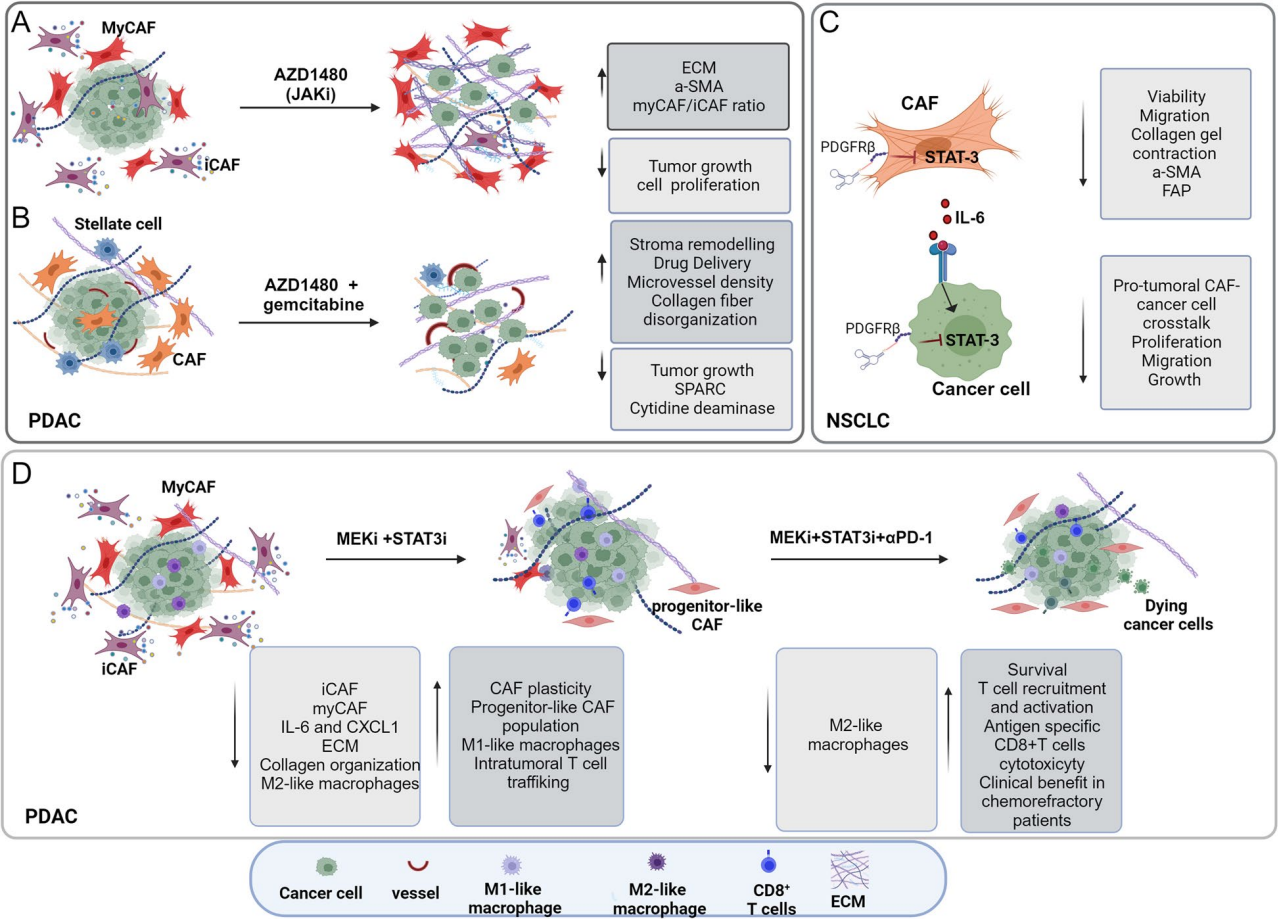
STAT3 signaling targeting modulates the stroma by affecting CAF activation/functions and cancer cell/CAF crosstalk in PDAC and NSCLC. Effects of the JAK/STAT axis targeting by JAK inhibitor AZD1480 on: (**A**) reprogramming inflammatory CAFs (iCAFs) in a mouse model of PDAC; (**B**) affecting in vivo drug delivery and therapeutic response in PDAC, in combination with gemcitabine [119, 120]. **C** Hampering CAF activity and the crosstalk between NSCLC cells and CAFs through STAT3 silencing by an aptamer-based strategy [121]; (**D**) Reprogramming of the CAF population and the immune microenvironment to overcome resistance to immune checkpoint blockade in PDAC by the combination of MEKi (trametinib) and STAT3i (ruxolitinib) [122]

Targeting STAT3, known as a key mediator of CAF pro-tumoral actions, offers an opportunity to indirectly target pro-tumoral CAF function, especially when combined with other therapies (Fig. 3). An interplay between PTEN and STAT3 has been identified in regulating pro-tumorigenic CAF functions and modulating the ECM production and composition in PDAC [118], suggesting that STAT3 inhibition could decrease the fibrotic stroma and limit immunosuppressive pathways.

As already discussed, JAK/STAT signaling maintains an inflammatory CAF phenotype through a positive feedback loop involving STAT3-mediated upregulation of IL1R1. Interestingly, treating tumor-bearing mice with the JAK inhibitor AZD1480 resulted in decreased cancer cell proliferation and tumor growth, increased collagen deposition, and in an apparent shift from an inflammatory CAF (iCAF) phenotype towards a myCAF-like state (Fig. 3A) [66]. The therapeutic potential of remodeling the ECM by targeting STAT3 signaling has been reported in a study indicating that AZD1480 inhibitor, when combined with gemcitabine, enhances drug delivery in PDAC models by effectively remodeling the tumor stroma [119], without detrimental effect [120] (Fig. 3B). Additionally, a recent research revealed the therapeutic applicability and efficacy of targeting STAT3 to inhibit CAF-NSCLC cell crosstalk using STAT3 siRNA delivery targeted systems based on nucleic acid aptamers as carriers. This strategy showed promise in hampering the pro-tumoral functions of CAFs and inhibiting the crosstalk between NSCLC cells and CAFs [121] (Fig. 3C).

In PDAC, STAT3 activation also plays a crucial role in mediating resistance to mitogen-activated protein kinase/extracellular signal-regulated kinase inhibition (MEKi). Remarkably, a combination of MEKi and STAT3 inhibition leads to in vivo stroma remodeling, partly mediated by CAFs, which reduces intratumoral immune suppressive cell infiltration, enhances CD8 + T cell trafficking, and overcomes resistance to immune checkpoint inhibitors such as programmed cell death protein 1 (PD-1) blockade in preclinical models. The combination of Trametinib (MEKi), Ruxolitinib (STAT3i), and Nivolumab (anti–PD-1) has shown clinical efficacy and tolerability in a patient with chemotherapy-refractory PDAC, offering promise for PDAC patients [122] (Fig. 3D). This combination is currently being evaluated in a phase 1 clinical trial for patients with chemo-refractory metastatic PDAC (NCT05440942).

Active clinical trials are examining the safety and efficacy of STAT3 inhibitors in combination with immunotherapies in various cancers. AZD9150 (Danvatirsen), an antisense oligonucleotide inhibitor of STAT3 mRNA expression, is being investigated in a clinical trial (NCT03421353) for patients with advanced solid tumors, including NSCLC, either alone or in combination with chemotherapy. Danvatirsen in combination with Durvalumab, a programmed death-ligand 1 (PD-L1) inhibitor, is currently being tested in a phase II clinical trial (NCT02983578) for patients with advanced and refractory pancreatic cancer, NSCLC, and Mismatch Repair Deficient Colorectal Cancer.

These findings demonstrate the clinical potential of targeting STAT3 in modulating the TME and enhancing the efficacy of other therapeutic approaches.

## Targeting Myc

The oncogenic TF c-MYC plays a crucial role in fibroblast transformation into CAF-like phenotype and its transcriptional networks have been involved in the trascriptome rewiring of metastasis-associated fibroblasts, associated with disease progression in human breast cancer [59].

Moreover, the MYC-driven secretion of exosomes containing miR-105 in breast cancer cells triggers a MYC-gene expression program in surrounding CAFs, leading to metabolic reprogramming that enhances glucose and glutamine metabolism to fuel adjacent cancer cells. This higher MYC activity in CAFs, required the down-regulation of MAX Interactor 1, Dimerization Protein (MXI1), a known antagonist of MYC transcriptional activity [123].

Several strategies, which include treatments that directly target c-MYC, or inhibit its translation or the interaction with the partner proteins, have been developed. Furthermore, the identification of novel c-MYC co-factors could represent new insights in the development of therapeutic strategies inhibiting c-MYC activity [124]. OMO-103, a direct c-MYC inhibitors, has recently entered in human clinical practice in combination with the standard regimen gemcitabine/nab-paclitaxel in treatment-naïve patients with metastatic pancreatic (NCT06059001).

Targeting c-MYC opens up new possibilities for therapeutic intervention, especially with the development of nanoparticle-mediated drug delivery strategies as revealed by αvβ3 integrin-nanoparticole-mediated drug delivery of a c-MYC inhibitor in breast cancer [125]. The selectivity of such approaches in targeting specific cell populations, as it has been reported for tumor-promoting M2 macrophages [125], may suggest that the selective target of c-MYC holds promise for more effective and tailored cancer treatments through targeting pro-tumoral cancer cell/CAFs crosstalk.

## Targeting EMT-TFs

As above reported EMT TFs play a major role in fibroblast activation and subtype definition. This could open up promising avenues for therapeutic interventions. Two small molecules inhibitors, GN25 and GN29, able to impede the interaction between p53 and SNAIL, have been shown to inhibit SNAIL mediated transcription [126]. Furthermore, targeted inhibition of SNAIL family of TFs by oligonucleotide-Co (III) Schiff base conjugate has been also achieved [127]. Additionally, in aggressive cancer cells, the small-molecule compound CYD19 has been shown to bind SNAIL, impairing its interaction with CREB-binding protein (CBP)/p300, which consequently impairs CBP/p300-mediated SNAIL acetylation and promoting its degradation through the ubiquitin–proteasome pathway [128]. Harmine, an alkaloid compound with anti-tumor activity in various oncogene-driven NSCLC types, has been shown efficacy in promoting TWIST1 protein degradation, suggesting a therapeutic strategy for treating oncogene-driven NSCLC [129]. However, thorough evaluations are necessary to determine the safety and efficacy of these molecules in clinical settings.

For targeting ZEB1, miRNA-based therapeutic options [130] and inhibitors of chromatin regulators, such as BET4 proteins which target the DNA endonuclease Mus81, which in turn regulates ZEB1 [131], have been proposed. Furthermore, small molecule inhibitors disrupting the interaction between CBFβ and RUNX exhibit the ability to inhibit colony formation in basal-like breast cancer cell lines, and to impair the growth of leukemia cell lines [132].

The local administration of LNA-modified oligonucleotides (ASOs) targeting PRRX1, specifically to the lung via an endotracheal route, has shown effectiveness in attenuating lung fibrosis in a mouse model [24]. This localized approach indicates the possibility of inhibiting mesenchymal transcription factors in specific cancer tissue context.

## Targeting of YAP and TAZ

YAP and TAZ, as primary mechanosensitive transcription factors, have gained attention due to their established roles in promoting cancer cell proliferation, migration, metastasis, and resistance to therapies, prompting exploration as potential therapeutic targets [133]. Initial efforts aimed to disrupt YAP/TAZ-TEAD binding, revealed Verteporfin, a benzoporphyrin derivative that inhibits liver cell growth [134]. Additionally, a specific TEAD inhibitor, palmitoylation MGH-CP1, disrupted the YAP-TEAD interaction, offering new insights into YAP-TEAD targeting [135].

Another approach involves a peptide mimicking mammalian Vestigial-like proteins (VGLL1-4), which acts as a YAP antagonist in gastric cancer by interfering with YAP-TEAD interaction and target gene expression [136]. An alternative strategy indirectly suppresses YAP and TAZ activity by modulating their upstream regulators. Dasatinib, a tyrosine kinase inhibitor, induces YAP phosphorylation, thereby suppressing YAP/TAZ-TEAD target gene expression in renal cell carcinoma [137]. Similarly, FAK inhibition shows potential in suppressing YAP activity for uveal melanoma treatment [138]. Furthermore, blocking YAP nuclear translocation is an additional therapeutic avenue. Norcantharidin and dobutamine have demonstrated the inhibition of YAP nuclear localization and its associated gene transcription in NSCLC and human osteoblastoma cells, respectively [139, 140]. Statins, via inhibition of HMG-CoA reductase, have been found to impede YAP/TAZ nuclear localization, attenuating established lung fibrosis in a mouse model [141].

The development of small molecule inhibitors directly against YAP and TAZ faces challenges due to the complexities of these molecules and the limited therapeutic inhibitory regions [133]. However, few clinical trials targeting YAP are currently ongoing. An antisense oligonucleotide against YAP is undergoing in a phase I trial for advanced solid tumors (NCT04659096), while an inhibitor targeting YAP/TAZ-mediated transcription is in a phase I clinical trial for advanced mesothelioma and other solid cancers (NCT04857372).

Targeting YAP and TAZ shows promise for affecting both cancer cells and stromal components, but further research is crucial to understand the mechanisms, dosage, duration, and efficacy of these proposed drugs, especially their potential impact on healthy tissues and immune regulatory functions in clinical settings.

## Conclusions

In summary, this review comprehensively outlines the pivotal TFs responsible for NF conversion into CAFs, their activation and the intricate crosstalk between CAFs and cancer cells. Phenotypical and functional heterogeneity is a pronounced feature of CAFs that clearly emerges from the transcriptomic analysis of different CAF subpopulations and from the identification of active TF regulatory networks and associated gene signatures. These findings facilitate the advancement of our knowledge of CAF biology and pave the way for a more precise characterization of CAF subtypes. Although a number of TFs and their associated potential therapeutic role have been suggested, further in-depth and comprehensive knowledge in this field is needed to identify new opportunities for more effective therapeutic strategies to selectively target the pro-tumorigenic CAF functions, while preserving normal fibroblasts.

## Data Availability

Not applicable.

## Abbreviations

TFs: Transcription factors
CAFs: Cancer Associated fibroblasts
NFs: Normal fibroblasts
TME: Tumor microenvironment
EN1: Engrailed-1
ECM: Extracellular matrix
RUNX1-2: Runt-related transcription factors 1 and 2
TCF4: Transcription Factor 4
ZEB1-2: Zinc-finger E-box-binding homeobox1 and 2
SOX2: SRY-Box Transcription Factor 2
EGR2: Early growth response 2
FOXS1: Forkhead box s1
PRRX1: Paired-related homeobox protein
BNC2: Basonuclin 2
TBX2-4-5: T-box transcription factors
ATF3: Activating transcription factor 3
AR: Androgen receptor transcription factor
HSF1: Heat-shock factor 1
YAP-TEAD: Yes-associated protein-transcriptional enhanced associate domain
MRTF-SRF: Myocardin-related transcription factor-serum response factor
HIF-1α: Hypoxia-inducible factor 1 alpha
TFAM: Mitochondrial transcription factor A
SALL4: Spalt-like transcription factor 4
ETV1: ETS1 variant transcription factor
MZF1: Myeloid zinc finger 1
MEF2C: Myocyte enhancer factor 2 C
myCAF: Myofibroblastic CAFs
iCAF: Inflammatory CAFs
apCAF: Antigen presenting CAFs
vCAFs: Vascular CAFs
mCAFs: Matrix CAFs
cCAFs: Cycling CAFs
dCAFs: Developmental CAFs
NSCLC: Non-small cell lung cancers
PDAC: Pancreatic ductal adenocarcinoma
CRC: Colorectal cancer
BLCA: Bladder cancer cells
SCC: Squamous cell carcinoma
HGF: Hepatocyte growth factor
FGF7: Fibroblast growth factor 7
